# The Glymphatic–Immune Axis in Glioblastoma: Mechanistic Insights and Translational Opportunities

**DOI:** 10.3390/ijms27020928

**Published:** 2026-01-16

**Authors:** Joaquin Fiallo Arroyo, Jose E. Leon-Rojas

**Affiliations:** 1 NeurALL Research Group, Quito 170157, Ecuador; joaquin.fiallo@udla.edu.ec; 2 Grupo de Investigación Bienestar, Salud y Sociedad, Escuela de Psicología y Educación, Universidad de Las América, Quito 170125, Ecuador

**Keywords:** glymphatic system, glioblastoma, brain tumor, glymphatic–immune axis, tumor microenvironment

## Abstract

Glioblastoma (GBM) remains one of the most treatment-resistant human malignancies, largely due to the interplay between disrupted fluid dynamics, immune evasion, and the structural complexity of the tumor microenvironment; in addition to these, treatment resistance is also driven by intratumoral heterogeneity, glioma stem cell persistence, hypoxia-induced metabolic and epigenetic plasticity, adaptive oncogenic signaling, and profound immunosuppression within the tumor microenvironment. Emerging evidence shows that dysfunction of the glymphatic system, mislocalization of aquaporin-4, and increased intracranial pressure compromise cerebrospinal fluid–interstitial fluid exchange and impair antigen drainage to meningeal lymphatics, thereby weakening immunosurveillance. GBM simultaneously remodels the blood–brain barrier into a heterogeneous and permeable blood–tumor barrier that restricts uniform drug penetration yet enables tumor progression. These alterations intersect with profound immunosuppression mediated by pericytes, tumor-associated macrophages, and hypoxic niches. Advances in imaging, including DCE-MRI, DTI-ALPS, CSF-tracing PET, and elastography, now allow in vivo characterization of glymphatic function and interstitial flow. Therapeutic strategies targeting the fluid-immune interface are rapidly expanding, including convection-enhanced delivery, intrathecal and intranasal approaches, focused ultrasound, nanoparticle systems, and lymphatic-modulating immunotherapies such as VEGF-C and STING agonists. Integrating barrier modulation with immunotherapy and nanomedicine holds promise for overcoming treatment resistance. Our review synthesizes the mechanistic, microenvironmental, and translational advances that position the glymphatic–immune axis as a new frontier in glioblastoma research.

## 1. Introduction

Glioblastoma (GBM) is the most aggressive and lethal primary brain tumor in adults, classified as a World Health Organization (WHO) Grade IV glioma [1]. Despite the standard treatment protocol, median survival is approximately 14–16 months, and the five-year survival rate is below 5% [1]. This aggressiveness and the almost inevitable recurrence are largely due to the limited infiltration of drugs across the barriers of the central nervous system (CNS) and the inherent adaptability of tumor cells [1].

Historically, the CNS was considered an “immunologically privileged” site due to the supposed absence of lymphatic vessels, which led to the belief that the cerebrospinal fluid (CSF) functioned primarily as a passive sink for metabolic waste [2]. However, this view has been revised. CNS homeostasis depends on specialized barriers, including the blood–brain barrier (BBB), composed of endothelial cells with continuous tight junctions (TJs) that strictly restrict the passage of substances; the blood–CSF barrier (BCSFB), located in the choroid plexus; and the glymphatic system [2]. The latter is a waste-clearance network that facilitates the entry of CSF into the parenchyma through the perivascular spaces surrounding the arteries, being facilitated by the water channel protein Aquaporin-4 (AQP4) located in astrocytic endfeet [3].

The pathology of GBM is characterized by marked cellular heterogeneity that allows adaptation and resistance [1]. The tumor microenvironment (TME) is highly complex and immunosuppressive, harboring a large number of tumor-associated macrophages/microglia (TAMs), which can constitute up to 50% of the cells, driving a pro-tumoral phenotype [1]. In this context, the central hypothesis is that dysfunction of the glymphatic and immune barriers co-contributes to the therapeutic resistance of GBM: glymphatic dysfunction and disruption of the BBB (often correlated with mislocalization of AQP4) restrict the penetration of therapeutic agents and compromise the drainage of tumor antigens toward the meningeal lymphatic vessels (MLVs) [4]. The emerging relevance of the glymphatic–immune axis is that drainage through the MLVs is crucial for immune surveillance, which underscores the fact that the brain is not completely isolated from the immune system, as previously thought [5]. This knowledge provides a vital clinical and translational foundation for the design of new strategies, as targeting fluid dynamics (for example, through the modulation of AQP4 or the use of the perivascular route for drug delivery) offers a novel target to potentially overcome GBM resistance [6].

Our review was developed using a structured narrative approach integrating mechanistic, microenvironmental, imaging, and therapeutic evidence. A comprehensive literature search was conducted in PubMed focusing on publications from 2012 to 2025 that examined glymphatic physiology, interstitial fluid dynamics, blood–brain and blood–tumor barrier biology, tumor–immune interactions, glioblastoma imaging, and emerging therapeutic strategies. Search terms included combinations of “glioblastoma”, “glymphatic system”, “interstitial fluid flow”, “aquaporin-4”, “blood–brain barrier”, “meningeal lymphatics”, “drug delivery”, “focused ultrasound”, “nanoparticle”, and “immunotherapy”. Additional references were identified through citation tracking of key papers. Peer-reviewed experimental, clinical, imaging, and translational studies were prioritized, including recent high-quality preclinical models and human imaging investigations. The selection and synthesis emphasized mechanistic consistency, reproducibility, and clinical relevance. This methodology ensured an integrated and up-to-date framework to characterize how fluid and immune barriers influence glioblastoma biology and therapy.

## 2. The Glymphatic System and CNS Fluid Dynamics

### 2.1. Architecture and Mechanisms

In the human body, the system responsible for filtering and eliminating waste molecules is the lymphatic system, which, through specialized vessels distributed throughout the body, collects this waste and deposits it into the central bloodstream. The exception is the CNS, which does not have a traditional lymphatic system but instead has a specific cleaning mechanism known as the glymphatic system (GS). The GS is composed of different anatomic, molecular, and functional parts. The anatomic pathways involved in this system are para-arterial pathways, paravenous drainage pathways and the interstitial space [7]. The main fluids that occupy these pathways are the CSF, interstitial fluids (ISFs), and interstitial solutes which have a key role in the fluid dynamics of the GS; astrocytes, astrocytic endfeet, and AQP4 are the three main molecular and cellular components [2]. Finally, arterial pulsatility together with the hydrostatic gradient compose the two driving forces of this system [8]. In the context of the glymphatic system, paravascular spaces refer to the fluid-filled compartments surrounding penetrating arteries and arterioles, through which cerebrospinal fluid (CSF) enters the brain parenchyma from the subarachnoid space [2,7,8]. These para-arterial pathways support CSF influx and are functionally driven by arterial pulsatility and pressure gradients. In contrast, perivascular spaces is a broader term encompassing vessel-associated pathways surrounding arteries, capillaries, and veins; in glymphatic physiology, perivascular efflux predominantly occurs along venous and intramural basement membrane pathways, facilitating interstitial fluid (ISF) and solute clearance from the brain [2,7,8]. While the terms are sometimes used interchangeably in the literature, in this review we use “paravascular” to denote CSF influx routes and “perivascular” to describe vessel-associated clearance pathways, reflecting their distinct functional roles within glymphatic transport.

Perivascular pathways play a key role in the GS allowing CSF influx and efflux; para-arterial pathways surround the penetrating arteries and arterioles of the cerebral parenchyma, these spaces (i.e., Virchow–Robin compartment) allow a massive influx of CSF from the subarachnoid space into the cerebral parenchyma [2]. The solute passage from the paravascular space into the surrounding tissue is size dependent, suggesting astrocytic endfeet play a filtering function [2]. On the other hand, ISF clearance is mediated by large caliber paravenous pathways surrounding the medial cerebral veins and caudal lateral rhinal veins [2]. The cerebrospinal fluid that enters in large amounts mixes with the interstitial fluid and solutes, a process known as bulk flow; this phenomenon occurs between the influx and efflux pathways [9]. Bulk flow is a critical component for the ISF solute elimination which includes the elimination of soluble β-amyloid (Aβ1-40), for example [2].

AQP4 is a water channel protein essential for fluid exchange in the central nervous system; it is highly polarized in the perivascular astrocytic endfeet surrounding the cerebral microvasculature which provides a low-resistance pathway for fluid movement between paravascular spaces in the interstitium of brain cells [10]. The massive influx of ISF drives the removal of interstitial solutes and is dependent on transinterstitial transport, which is mediated by AQP4. In an in vivo study with mice, it was shown that the elimination of interstitial solutes was reduced by 70% when the AQP4 gene was eliminated, demonstrating the critical role of AQP4 in solute elimination from the ISF [2]. This polarization is dependent on the blood vessel; other studies have shown that periarterial astrocytic endfeet have a lower reactivity to AQP4 compared to perivenous or pericapillary ones [11]. Transglial H_2_O transport is driven by the hydrostatic pressure of the para-arterial bulk flow and arterial pulsatility; these two forces are the primary drivers of CSF flow [8,11]. Arterial pulsatility, along with the low resistance of the venous sinuses, creates an arteriovenous hemostatic gradient that drives solute flow from the perivascular space into the interstitium [10,11]. Differences in AQP4 expression contribute to polarization and flow along the efflux and influx pathways; for example, studies have proposed that sleep plays an important role in the elimination of macroscopic waste products thanks to an expansion of the extracellular space induced by a reduction in noradrenergic signaling that occurs during the slow-wave phase [11]. Certainly, an in vivo study using mice demonstrated an increase in waste clearance of up to 60%, thanks to the expansion of the interstitial space, leading to a reduction in flow resistance and increase in CSF exchange and movement [11].

### 2.2. Alterations in a Tumoral Context

Brain tumors cause distortions in the GS and in CSF dynamics within the perivascular spaces for several reasons. First, the tumor mass produces an anatomical distortion that can lead to astrocytic displacement of AQP4 in peritumoral areas; this phenomenon is known as tumor mass effect [4]. Tumor mass effect compromises the glymphatic pathways by distorting the perivascular spaces and compressing the CSF-ISF exchange zones [4]. The growth of the tumor mass may cause secondary increases in the intracranial pressure which can obstruct CSF flow. In a study conducted on rats using tracers, it was observed that glioblastoma caused low CSF flow rate and decreased bulk flow; tumor growth, in turn, obstructed the drainage and clearance of the GS, causing tracer accumulations in the cerebellum and total blockage of tracer clearance in the CSF cisterns in some of the experimental subjects [3]. Glioblastomas also cause alterations in AQP4 protein distribution and polarization, instead of concentrating in astrocytic endfeet, polarized AQP4 starts redistributing throughout the entire astrocyte cell membrane, suffers upregulation and results in a disorganized polarization which leads to an augmentation of the hematoencephalic barrier permeability and exacerbation of vasogenic edema [4]. AQP4 alteration is closely related to peritumoral edema as it creates abnormal osmotic gradients that contribute to the aberrant influx of water into the extracellular space, which affects the tumoral microenvironment.

Furthermore, glioma stem cells (GSCs) secrete tenascin-C (TNC) that together with the acidic environment, resulting from the incremented anaerobic glycolysis generated by tumor cells, promotes the migration of malignant cells and the increase in angiogenic factors [1]. The GS is crucial to clear these factors secreted or indirectly stimulated by tumor cells, so the described alterations in its function can cause the accumulation of these toxic and pro-inflammatory molecules, including cytokines and chemokines that can further worsen the tumor microenvironment fostering growth, migration, and survival of the tumor cells [1,4,5]. This system also plays an important role in the distribution of drugs and antitumor agents; for example, in therapies such as carmustine wafers (BCNU), which are surgically placed directly into the brain, the drug concentrations reaching the deep regions of the brain parenchyma are very limited, contributing to its limited therapeutic effectiveness [5]. Increased intracranial pressure has also been observed to hinder drug therapies by reducing blood flow, which in turn reduces the distribution of systemic drugs [5].

## 3. Immune and Structural Barriers Interacting with Fluid Dynamics

The central nervous system has long been considered to have an immunological privilege and isolation that depends on highly specialized barriers together with fluid drainage networks. Recent studies have challenged this notion of a completely isolated brain by revealing pathways that control both the entry of immune cells into the CNS and the elimination of waste and antigens [12]. The BBB is one of the crucial structures for maintaining this homeostasis in the central nervous system; it is a highly specialized vascular structure that regulates everything that enters and exits the systemic circulation into the CNS, maintaining an optimal microenvironment for neuronal function [13]. The integrity and filtering capacity of this barrier depends on several components, especially on the endothelial cells of the brain capillaries; these cells are mainly distinguished by low pinocytic activity, high mitochondrial volume, and the presence of tight junctions (TJs), which are formed by proteins such as claudin-1, claudin-5, occludin, and zonula occludens proteins (ZO-1, -2, -3) that together seal the paracellular space and confer high electrical resistance [14]. In glioblastoma, for example, the BBB is altered, giving rise to a blood–tumor barrier (BTB). Proteins such as ZO-1 and claudin-5 are affected, leading to a loss of tight junctions, which induces a phenomenon known as a leaky BBB; this allows the extravasation of blood-borne molecules not only into the tumor mass, affecting its microenvironment, but also into the central nervous system as a whole [15].

**Figure 1:**
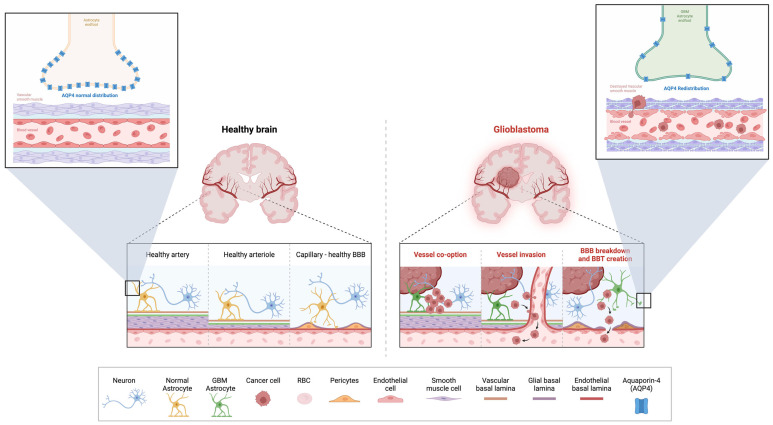
**Comparative Architecture of Vascular, Astrocytic, and Aquaporin-4 Dynamics in the Healthy Brain versus Glioblastoma.** This figure compares the structural and molecular organization of neurovascular and glial interfaces in the healthy brain (**left**) with the pathological alterations observed in glioblastoma (**right**). In healthy tissue, intact endothelial tight junctions, organized vascular and glial basal laminae, and astrocytic endfeet with polarized aquaporin-4 (AQP4) maintain BBB integrity and support glymphatic fluid exchange. In glioblastoma, tumor cells co-opt and invade vessels, disrupt smooth muscle layers, and displace astrocytic endfeet, leading to AQP4 depolarization, basement membrane degradation, BBB breakdown, and formation of a heterogeneous blood–tumor barrier. These changes promote edema, impaired fluid regulation, and a permissive microenvironment for tumor progression. Created in BioRender. Américas, U. (2026) https://BioRender.com/vlzdbo0 (accessed on 11 January 2026).

As previously stated, astrocytic endfeet play a key role in CNS fluid dynamics. Glioblastoma causes an invasion of malignant cells into the perivascular space that physically displaces the astrocytic endfeet from endothelial cells. A single glioma cell is enough to open the BBB and disrupt the normal astrocyte–vascular coupling and the basement membrane (BM) [15]. The latter is a laminar structure, 100–150 nm thick, produced by endothelial cells, astroglia, pericytes, and smooth muscle cells; this membrane has two layers: the endothelial BM and the astroglial BM [16]. Glioma tumor cells can degrade this membrane, causing further damage to tight junctions and increasing the permeability of the BBB [16]. Furthermore, AQP4, which is normally polarized in the astrocytic endfeet, shows a dysregulated expression and disorganization at the molecular level in the presence of gliomas. It gets redistributed across the astrocytic membrane in a form that is not associated with its typical orthogonal array of particles (OAPs) distribution. The disorganization facilitated by matrix metalloproteinases (MMPs), which degrade components such as dystroglycan and agrin, promotes vasogenic edema in the central nervous system in the presence of tumors [16]. Pericytes are another cellular lineage affected in glioblastoma; studies have shown that glioma cells invade along the vessels and envelop the pericytes [17]. Deficiencies and mutations in Notch3 have also been reported, which inhibit pericyte expression and compromise the integrity of the BBB [17]. To illustrate the contrasting structural and molecular features that underpin glymphatic and vascular integrity in health versus glioblastoma, Figure 1 provides a comparative overview of neurovascular architecture, astrocytic organization, and AQP4 localization under both conditions.

Drainage of CNS fluids toward the lymphatic system occurs through two mechanisms: drainage towards the dural lymph nodes and perivascular crosstalk. As previously described, the CNS parenchyma lacks conventional lymph nodes, but recent studies have confirmed the existence of functional lymphatic vessels in the dura mater, which assist in CNS drainage. Cerebrospinal fluid (CSF) drains from the subarachnoid space to the cervical lymph nodes through lymphatic nodes located in the nasal mucosa at the cribriform plate, along the cranial and spinal nerves via the dural lymphatics [12]. This is the main pathway that allows the transit of antigen-presenting cells (APCs), transporting CNS antigens to the peripheral immune system [12]. On the other hand, the interstitial fluid of the brain parenchyma uses another restricted pathway; it drains along the intramural basement membranes within the capillary walls of cerebral arterioles and arteries [12]. This pathway does not allow the entry of cells such as APCs, which represents a key anatomical restriction for the immune privilege of the brain parenchyma [12]. The second system is the perivascular crosstalk, which mediates the access of immune cells to the brain parenchyma and is controlled by two steps. The first step is the BBB, through which T cells cross the endothelium of post-capillary venules by receptor-mediated mechanisms, including alpha-4, beta-1 integrin, and endothelial VCAM-1. The endothelium of the BBB becomes activated during inflammatory processes, expressing adhesion molecules (VCAM-1, ICAM-1) and certain chemokines that act as promoters for leukocyte migration [14]. The second step of the perivascular crosstalk system involves the movement of T cells toward the brain parenchyma requiring them to cross a second barrier, the glia limitans [12]. Before crossing it, in the perivascular space, a checkpoint forces activated T cells to recognize antigens presented by perivascular APCs before entering the brain parenchyma; this process depends on the expression of matrix metalloproteinases (MMPs), including MMP2 and MMP9 [12].

The interaction of tumor cells with these barriers has effects that significantly influence the immune response. First, antigen clearance and meningeal lymphatics are affected, since the meningeal lymphatics disrupt the immunity cycle against GBM by facilitating the trafficking of antigens in APCs to the deep cervical lymph nodes, which in turn increases activation through the primary priming of T cells [18]. Following this, there is an activation of the pericyte–macrophage crosstalk that generates an immunosuppressive effect because, in the glioblastoma tumor microenvironment (TME), pericytes act as mediators of immunosuppression [19]. These pericytes display macrophage-like immune functions and regulate leukocyte trafficking [19]. In the context of GBM, they interact with tumor-associated macrophages (TAMs) and induce polarization toward a pro-tumoral, immunosuppressive M2 phenotype through the PDGF-BB/SOX7/IL pathway [18,19]. These interactions lead to the secretion of anti-inflammatory molecules (IL-10 and TGF-beta), which further increase the immune evasion of glioblastoma [19]. Finally, there is an induction of lymphatic remodeling mediated by VEGF-C, which has been shown to increase the efficacy of immune checkpoint inhibitors such as anti-PD-1/CTLA-4 [18].

To summarize the integrated physiological, vascular, and immunological disturbances described above, Table 1 outlines the principal mechanistic disruptions of the glymphatic–immune axis in glioblastoma together with their downstream consequences and therapeutic implications.

## 4. Imaging and Experimental Models

The effects on fluid dynamics caused by GBMs in the central nervous system require advanced imaging techniques and experimental models that enable noninvasive visualization of tumor pathophysiology. In this section, we discuss the preclinical tools and the traditional clinical imaging approaches used in GBM.

### 4.1. Preclinical Models

Preclinical experimental models are based on orthotopic models, which aim to replicate the invasiveness and characteristics of a human tumor by using human tumor cells implanted into the brains of immunosuppressed rodents [20]. Orthotopic xenograft models have allowed implanted patient-derived GSC stem cells (GSC1 and GSC275) to induce the development of highly infiltrative brain tumors, enabling the analysis of the interactions between tumor cells and the BBB [20]. One study reported that the BBB was preserved in most vessels located outside the main tumor; but they also noted that cells that disseminate and invade more distant areas use these blood vessels for migration [20]. Cell lines such as U87MG have also been used in these xenografts, but they showed poor capacity for brain invasion, which does not accurately replicate the clinical scenario observed in humans [20]. The synchronous models (GL261 cells) are the second type of orthotopic models used in the study of glioblastoma; these models are based on the implantation of murine glioma GL261 cells into immunocompetent mice such as C57BL/6 [21]. The advantages of using the murine glioma cell model are that it has been shown to replicate most of the characteristics of human GBM, presenting similarly rapid and aggressive growth patterns [22]. Furthermore, this model uses C57BL/6 mice, which are immunocompetent, unlike xenograft models that require immunosuppressed mice. However, its main feature is that GL261 GBM tends to grow as a bulky mass with limited invasive potential compared to the infiltrative nature of human GBM [3].

### 4.2. Fluid Tracing Techniques

Studies of CNS fluid dynamics are based on the intrathecal infusion of tracers or contrast agents into the cisterna magna. These studies are used to observe the movement of the tracers through the cranial compartments and towards the brain parenchyma [23]. An example of these is the use of fluorescent dextrans such as FITC-Dextran (5 kDa), which allows visualization of clearance, or Texas Red dextran (70 kDa), which allows visualization of the vascular system under microscopy [23]. Techniques such as epifluorescence microscopy are also used, in which 100-μm-thick coronal brain sections are obtained in ex vivo models, and two-photon fluorescence microscopy, which is used in vivo to analyze the penetration of intracisternal tracers [23]. The main limitation of the epifluorescence model is that it requires sacrificing the animal, since histological brain sections must be prepared [23]. Another technique used is dynamic contrast-enhanced magnetic resonance imaging (DCE-MRI), which employs paramagnetic contrast agents such as gadolinium that are administered directly into the CSF. The function of these contrast agents is to shorten the T1 relaxation time, allowing the tracking of solute and fluid transport within the glymphatic system [24]. A study conducted using these MRI techniques demonstrated that a monoclonal IgG1 antibody (ABT-806, 150 kDa) reached deep cortical and subcortical layers in rats, confirming that CSF pathways are a viable system for delivering drugs with large molecular sizes [25]. Intracerebral injections used to administer tracers have certain limitations that must be considered; intracranial pressure must be kept within normal limits during the infusion, as increased infusion rates can alter the rapid distribution of the contrast agent and disrupt normal CSF flow [24].

### 4.3. Traditional Clinical Imaging

DCE-MRI is also used to characterize vascular permeability and detect leakage in blood vessels. Gadolinium contrast is injected intravenously, and the enhancement of this contrast on T1-weighted images indicates areas where the BBB is disrupted [26]. This provides a pharmacokinetic parameter that may help predict a patient’s response to treatment [27]. In the context of the tumor microenvironment, it helps delineate tumor boundaries. Because it is a contrast-based study of the vascular system, it allows visualization of increased vascularization, which typically corresponds to the malignant area of a tumor [20]. Also, the combination of DCE-MRI with flow-vector analysis makes it possible to quantify the velocity, flow, and direction of interstitial fluid within the glioblastoma itself and in the surrounding regions [26]. Studies have shown that the areas surrounding the glioblastoma do not exhibit gadolinium enhancement, which suggests a well-preserved BBB, even though histological sections reveal invasion of tumor cells in that region [20].

The second technique used in traditional clinical imaging is diffusion tensor imaging along perivascular spaces (DTI-ALPSs). This is a non-invasive index that can serve as a marker of glymphatic function and interstitial fluid diffusivity [28]. It is based on the concept that perivascular CSF flows along the medullary venules, perpendicular to the projection and association fibers of the white matter [28]. It has been shown that DTI-ALPS has high reproducibility [29], and its use in patients with glioblastoma revealed a significantly decreased DTI-ALPS index compared with healthy individuals. There is an inverse correlation between the DTI-ALPS index and CSF volume, indicating a reduction in glymphatic function that affects fluid balance in patients with glioblastoma [28].

The last technique used for traditional clinical imaging is intrathecal contrast-enhanced magnetic resonance imaging. This technique involves administering gadolinium contrast intrathecally, usually in the lumbar region [24]. It has allowed visualization of glymphatic drainage toward the cervical lymph nodes, and it has also been observed that contrast injected via the spinal route enters the visual pathways [29]. What limits the use of this study is that contrast administration must be performed through an invasive method (potentially resulting in complications), and the scan times are very long, which restricts its use in fragile patients or in routine clinical settings.

### 4.4. Emerging PET and MRI Imaging Techniques

Positron emission tomography (PET) has been introduced in recent years as an alternative method that helps explore the pharmacokinetic aspects of CSF transport into the brain parenchyma and its efflux pathways. The activity of ^18^FDG can be used to quantify CSF transport and drainage toward different areas, such as the nasal turbinates [24]. New magnetic resonance techniques have also been developed, such as magnetic resonance elastography (MRE) with tomoelastography, which is a non-invasive method that allows quantification of the viscoelastic properties of tissue using the shear wave speed (SWS) and the loss angle [21]. This is useful in tumors because glioblastoma exhibits softer tissue properties than healthy brain tissue, which is attributed to pronounced vascularization and a high content of glycosaminoglycans (GAG) [21]. Multiparametric MRI has also been implemented for the generation of quantitative maps, such as proton density (PD) and quantitative susceptibility mapping (QSM). These techniques reflect water distribution and can show a positive association with global glymphatic function and a negative association with AQP4 expression in peritumoral edema [28]. This could be used together with DTI-ALPS to link glymphatic functionality with AQP4 levels in GBM patients. Finally, the last emerging imaging technique that has shown usefulness for GBM is interstitial fluid flow (IFF) vector analysis using DCE-MRI. Advanced computational models such as Regularized Optimal Mass Transport (rOMT) or Lymph4D are used to derive vector metrics of fluid movement from DCE-MRI images in in vivo models [26]. This technique quantifies the magnitude of flow velocity, the diffusion coefficient, and the density of trajectory lines originating from the tumor. The results obtained in this study using murine models suggest that higher flow velocity and a high density of tumor-originating pathlines correlate positively with, and predict, GBM invasion and progression, while a lower diffusion coefficient also correlates with tumor progression [26].

An emerging translational question is whether baseline or early treatment imaging of glymphatic function and neurofluid dynamics could serve as predictive or stratification biomarkers for response to immune checkpoint inhibitors (ICIs) in glioblastoma. Effective anti-tumor immunity within the central nervous system depends on adequate drainage of tumor antigens and immune cell trafficking through meningeal lymphatic pathways, and experimental evidence indicates that impaired meningeal lymphatic function suppresses, whereas lymphatic enhancement augments responses to anti-PD-1 and anti-CTLA-4 therapies in brain tumor models [6,18]. In this context, imaging-derived metrics that approximate cerebrospinal fluid–interstitial fluid (CSF–ISF) exchange may act as physiologically informed proxies of antigen drainage capacity and immune priming efficiency; candidate measures include the DTI-ALPS index and related diffusion markers of perivascular fluid movement [3,7,29], dynamic contrast-enhanced MRI–derived interstitial flow metrics and computational transport models that quantify spatial heterogeneity of fluid dynamics within and around the tumor [5,26,27], as well as CSF tracer kinetics assessed using intrathecal contrast MRI or PET-based CSF transport approaches [24,29]. Baseline stratification is biologically plausible, as marked glymphatic dysfunction, elevated intracranial pressure, and impaired CSF outflow have been demonstrated in glioma settings and may affect antigen egress toward cervical lymph nodes [4,5,18]. Moreover, early treatment-associated changes in edema burden, tissue biomechanics, or interstitial transport parameters could reflect partial restoration of fluid drainage and immune access, whereas persistently impaired metrics may identify an immune-refractory microenvironment despite therapy [4,5,18]. At present, however, direct clinical validation linking glymphatic imaging features to ICI response in glioblastoma remains limited, and these measures should be regarded as exploratory biomarkers requiring prospective evaluation alongside molecular profiling and immunotherapy outcomes [1]. To consolidate the methodological approaches discussed in this section, Table 2 summarizes the principal imaging and experimental tools used to characterize glymphatic function, fluid dynamics, and immune interactions in glioblastoma.

## 5. Therapeutic Implications

### 5.1. Drug Delivery Across the BBB

Glioblastoma and its high complexity require therapeutic plans to address several challenges, such as the intrinsic resistance of tumor cells, the difficulty of overcoming fluid barriers, and the immunosuppression of the tumor microenvironment. Drug delivery across the BBB is the main challenge in glioblastoma treatment, as it restricts the entry of most systemic pharmacological agents [30]. For this reason, several methods have been developed to evade, force, or modulate this barrier. Convection-Enhanced Delivery (CED) is a technique that uses direct intra-tumoral infusion, driven by a hydrostatic pressure gradient which propels the bulk flow of the drug into the extracellular space, bypassing the BBB [30]. A Phase 1 study of recurrent GBM showed that concentrations of the HDAC inhibitor entinostat in the perivascular niche were 20 times higher using CED compared with intravenous administration [31]. CED of nanoliposomal irinotecan demonstrated superior antitumor activity in intracranial GBM xenografts, and it also showed that the use of arborizing catheters can improve dispersion [31]. However, results are still mixed due to inadequate drug distribution in clinical trials as co-infusion of gadolinium for monitoring may not always reflect the actual drug distribution, and performing the technique is invasive [6]. Nonetheless, this method makes it possible to achieve high local concentrations in areas that regular systemic therapy cannot penetrate, making it a promising approach for the delivery of localized drugs and therapies.

Intrathecal (IT) administration bypasses the BBB by delivering pharmacological agents directly into the CSF, often through Ommaya implanted under the scalp. This route is viable for administering large-molecule drugs (IgG1, 150 kDa) into deep cortical and subcortical layers [6]; It has been used for the administration of liposomal cytarabine in leptomeningeal dissemination of CNS tumors [31]. Its main limitation is the rapid clearance of CSF, and the poor penetration of CSF into the brain parenchyma, which limits drug distribution [32]; additionally, it’s an invasive technique that requires surgery and may be associated with morphological changes [31].

Intranasal (IN) drug administration is another therapeutic alternative that can help drugs bypass the BBB. It has demonstrated rapid transport of tracers along perivascular spaces after intranasal administration. One study showed that IN delivery, specifically nanoliposomal SN-38, was an effective strategy in preclinical DMG models [33]. It has been observed that lymphatic clearance may be a potential limitation for this delivery method, which is still under investigation [33]. Nevertheless, this route could provide a non-invasive way to deliver certain agents to the CNS.

### 5.2. Nanoparticle (NP) and Liposomal Applications

**Figure 2:**
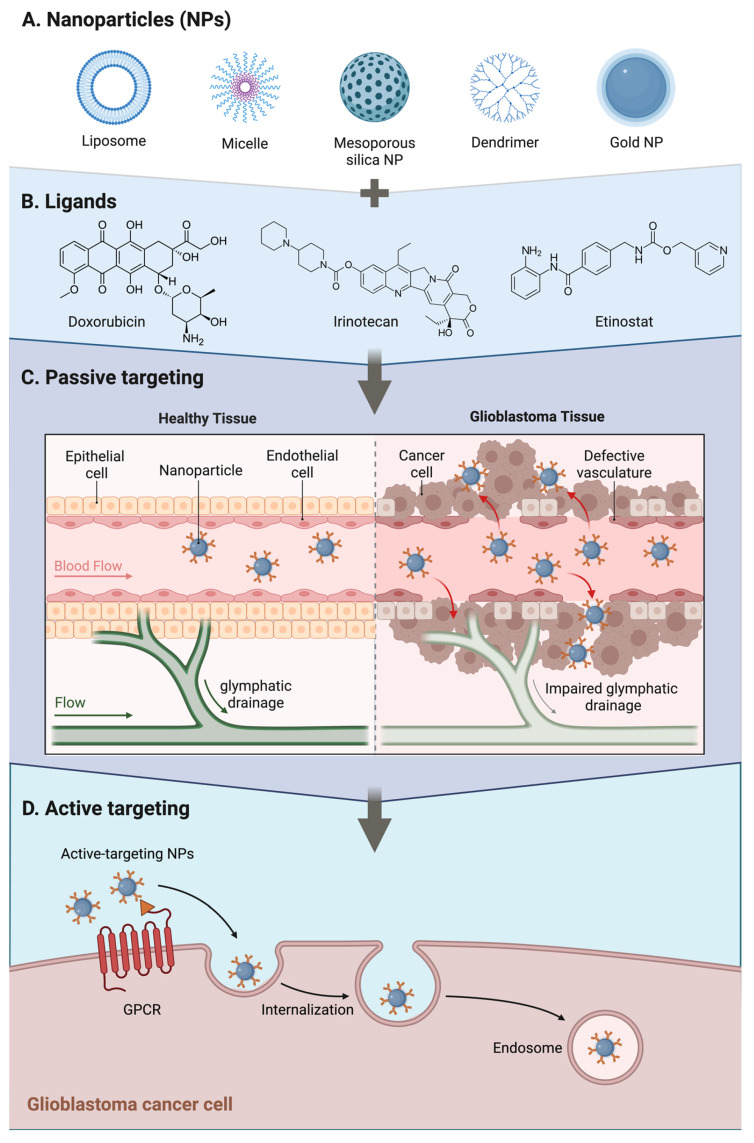
Nanoparticle Drug Delivery Systems for Glioblastomas. Created in BioRender. Américas, U. (2026) https://BioRender.com/97in8yc (accessed on 11 January 2026).

The use of nanoparticles for GBM therapies is based on the idea that nanoparticles aim to overcome the BBB through transcellular transport, either via receptor-mediated transport (RMT) or adsorptive-mediated transport (AMT) as shown on Figure 2; their size is essential for trafficking and tissue retention [31]. Nanoparticle systems are typically designed with sizes ≤ 100 nm for therapeutic use. One study used mesoporous silica nanoparticles (MSNs) of approximately 60 nm to load the STING agonist cdGMP, and cationic liposomes administered intra-arterially improved their deposition when assisted by transient cerebral hypoperfusion [34]. In another study, transferrin-conjugated nanoparticles (targeting the TfR1 receptor) loaded with GSK620 increased drug delivery to the brain by 4.8-fold in invasive cells [35]. HDL-mimetic nanoparticles have also been designed to specifically target medulloblastomas; these strategies have shown usefulness in encapsulating chemotherapeutic agents such as doxorubicin into polymeric nanoparticles for glioblastoma [36]. The main limitations of nanoparticle therapy are that penetration is hindered by the high intra-tumoral pressure and tumor-induced tissue stiffness, both of which affect drug retention within the parenchyma; the heterogeneity of the BTB and the presence of efflux pumps such as P-gp further reduce the effectiveness of these drugs [32]. However, the use of functionalized nanoparticles is key for active targeting; these particles deposit within the perivascular space and can be directed toward antigen-presenting cells (APCs) which makes them ideal candidates for future research endeavors [34].

The BBB/BTB can be transiently affected through physical forces or osmotic agents, such as arterial pulsatility or AQP4. As explained, the glymphatic system and its role in glioblastoma are largely modulated by fluid dynamics and by AQP4 [37]. BBB disruption is a technique that uses intra-arterial mannitol resulting in an increase in BBB permeability, allowing greater drug uptake in the tumor. This technique has been used to facilitate the delivery of agents such as methotrexate and bevacizumab [31]. The use of focused ultrasound (FUS) combined with microbubbles can also induce a temporary and noninvasive disruption of the tight junctions (TJs) of the BBB. This can enhance the delivery of systemic drugs such as carboplatin and doxorubicin in GBM models [38]. Finally, inhibition of RAGE using azeliragon (AZG) is currently under investigation in phase 1 and 2 clinical trials as an adjuvant therapy combined with chemoradiation for GBM. It has shown the ability to reduce cellular proliferation (Ki67^+^) in patient-derived tumor fragments [39]. These models have shown effectiveness; however, all of them have limitations. The BBB disruption model is an invasive method that requires intra-arterial catheterization and carries a wide range of adverse effects [31]. The FUS model is limited by the rapid closure of the BBB after treatment, which requires optimization for safety and continuous monitoring of drug delivery [6]. The AZG model, despite its good safety profile and its ability to penetrate the BBB, failed in phase 3 clinical trials for Alzheimer’s disease [39]. The transient effect created by BBB disruption or FUS provides a key therapeutic window for the efficient administration of systemic drugs to the tumor’s microenvironment, showing particular usefulness in patients who are in the postoperative period [40].

### 5.3. Immunomodulation and Clearance Enhancement

Glioblastoma is a highly resistant tumor due to profound immunosuppression and the presence of hypoxic niches in the tumor microenvironment that sequester and reprogram immune cells [34]. Meningeal lymphatic drainage provides a crucial route for the elimination of antigens from the CNS toward the cervical lymph nodes. It is also an essential pathway for the priming of T-cell responses and for immune surveillance against the tumor [33]. A study demonstrated that stimulation of this dural lymphatic drainage using VEGF-C in glioma models increased the efficacy of immune checkpoint inhibitors such as anti-PD1 and CTLA-4 [6]. Photodynamic therapies induce immunogenic cell death (DAMPs) and enhance the clearance of waste products by stimulating the meningeal lymphatic vessels [33]. The use of pharmacological agents such as exogenous interleukin-33 (IL-33) has also been documented, showing improvements in cerebral lymphatic drainage and in the clearance of toxic proteins in mouse models with traumatic brain injuries [41]. These therapies help restore lymphatic drainage and enhance systemic immunotherapy by facilitating the presentation of tumor antigens [33]. However, the limitations of these therapies are mediated by tumor growth, as it can obstruct the lymphatic outflow of the CSF, thereby hindering drainage [6]. PDT therapies require preclinical and clinical studies to help optimize treatment regimens and understand the underlying immunological mechanisms.

Tumor-associated macrophages and microglia (TAMs) are the most abundant immune cell population within the glioblastoma tumor microenvironment. They are attracted to hypoxic regions typically where pseudopalisading tumor cells are located and become reprogrammed into an immunosuppressive phenotype. This phenomenon involves several factors, including niche-derived signals such as CCL8 and IL-1β [42]. Inhibition of CSF-1R with BLZ945 has been shown to block glioma progression; however, this therapeutic approach leads to fibrotic scarring driven by TGF-β and IL-1β, which creates pro-tumoral niches that support recurrence [43]. To prevent this, a combination therapy of BLZ945 + Galunisertib (a TGF-β inhibitor) is used, which has shown significant improvements in survival in preclinical studies by inhibiting the fibrosis associated with the treatment [43]. Therapy with STING agonists has also been used in cases of GBM. The STING agonist cdGMP reprograms macrophages toward an anti-tumor phenotype, and treatment with immuno-MSN (cdGMP) was observed to delay tumor growth and increase dendritic cells (CD11c+) and circulating CD8+ T cells [44]. Finally, SMAC mimetics (GDC-0152) reduce the number of pro-tumoral P5 macrophages and modulate the microglial phenotype. Local administration of a chemotherapeutic hydrogel called GemC12-LNC, in combination with postoperative systemic administration of GDC-0152, significantly increased survival (32 days vs. 42 days, *p* = 0.0027) in one study [40].

From a clinical standpoint, glymphatic dysfunction in glioblastoma is unlikely to represent a binary exclusion criterion for immunotherapy, but rather a continuous modifier of treatment efficacy. Mechanistic and imaging evidence discussed throughout this review supports a graded model in which progressive impairment of CSF-ISF exchange, increased intracranial pressure, AQP4 depolarisation, vasogenic edema, and restricted meningeal lymphatic drainage incrementally limit tumor antigen egress and immune priming, thereby reducing the likelihood and magnitude of response to immune checkpoint inhibitors [4,5,6,18,45,46]. Within this framework, glymphatic function exists along a physiological continuum and interacts with tumor burden, vascular permeability, tissue biomechanics, and treatment-induced changes rather than acting as an on-off determinant of therapeutic eligibility [1,3,5,21]. Importantly, experimental studies demonstrating that partial restoration or enhancement of meningeal lymphatic drainage can improve antitumor immune responses support the concept that glymphatic impairment is modifiable and potentially exploitable as an optimisation parameter, rather than a contraindication for immunotherapy [18,46]. Framing glymphatic dysfunction as a dynamic and quantitative modifier preserves clinical flexibility while reinforcing its relevance for patient stratification, treatment timing, and rational combination strategies.

Finally, radiotherapy exerts dual and temporally dynamic effects on the glymphatic–immune axis in glioblastoma, with both potentially beneficial and detrimental consequences for antitumor immunity. In the acute and subacute phases, radiotherapy can reduce tumor burden and intracranial pressure, induce immunogenic cell death with release of damage-associated molecular patterns (DAMPs), and transiently modify vascular permeability, thereby increasing antigen availability and facilitating immune priming through meningeal lymphatic pathways [1,5,18,33]. These early effects may coincide with partial normalisation of the tumor microenvironment, creating a permissive state for immune activation and providing a potential window of opportunity for synergy with immune checkpoint inhibitors [18,47,48]. In contrast, delayed and cumulative effects of radiation include vasogenic edema, endothelial dysfunction, astrocytic injury with AQP4 depolarisation, and progressive extracellular matrix remodelling and fibrosis, all of which are expected to impair perivascular and paravascular fluid transport and compromise long-term glymphatic and lymphatic drainage [4,5,8,14,37]. These changes may restrict antigen egress and immune cell trafficking, thereby attenuating sustained immunotherapeutic efficacy; importantly, this biphasic response underscores the time-dependent nature of radiotherapy-immune interactions, suggesting that optimisation of treatment sequencing and timing is critical [4,5,8,14,37]. Aligning immunotherapy delivery with early post-radiotherapy intervals characterised by enhanced antigen availability and relatively preserved fluid transport may maximise therapeutic benefit, whereas delayed administration may encounter a progressively immune-refractory and fluid-restricted microenvironment [1,5,18]. Given the breadth of GBM immunotherapy, we refer readers to comprehensive syntheses covering checkpoint blockade, vaccines, myeloid targeting, and the immune microenvironment, and to focused reviews on meningeal lymphatics as regulators of antigen drainage and therapeutic response [45,46,47,48].

## 6. Challenges, Knowledge Gaps, and Future Directions

The difficulty to precisely model the tumor microenvironment (TME) of GBM and its heterogeneity is partly responsible for the failure in translating basic research into successful clinical therapies [1]. The most commonly used animal models, such as patient-derived xenografts (PDXs) implanted in immunodeficient mice, do not incorporate the full immune complement of GBM. Furthermore, extrapolating findings from rodents to humans requires caution due to substantial differences between mouse and human brains, including their structure and functional organization, as well as evolutionary genetic differences [18]. In the context of fluid dynamics, drainage through nasal lymphatics and perineural pathways may be less pronounced in humans compared to rats; it has also been reported that contrast distribution in the brain is slower in humans [24].

The study of glymphatic flow in patients is hindered by technical limitations. The most direct methodologies, which involve intrathecal injection of tracers (such as gadolinium), are invasive and therefore unsuitable for broad adoption in human in vivo studies [7]. Non-invasive approaches have focused predominantly on techniques such as the DTI-ALPS index. However, it has significant limitations as it evaluates diffusion along perivascular spaces (PVSs) in white matter, whereas glymphatic movement primarily involves the interstitium in gray matter [5]. Consequently, the precise correlation between DTI-ALPS measurements and glymphatic functionality remains intricate and poorly understood. It is known that these measurements are affected by imaging plane and head position [29].

At a mechanistic level, significant uncertainties persist in understanding the glymphatic system. The mechanisms driving solute and waste clearance from the interstitial space along perivascular pathways are not yet fully elucidated. Moreover, the precise and complete role of AQP4 in perivascular fluid transport remains elusive [49]. A central challenge is the lack of clarity regarding the temporal coupling between CSF dynamics, immune trafficking, and tumor progression. The exact association between the glymphatic system and GBM cell infiltration remains unknown [3]. Likewise, how CNS-derived antigens and immune cells exit the CNS and alter meningeal lymphatic function continues to be incompletely understood.

To overcome these challenges, the future of research depends on the need for multiscale models that incorporate all aspects of the tumor microenvironment; this involves deep integration of in vitro techniques, such as the development of BBB/glymphatic-on-a-chip barrier models, with dynamic in vivo approaches that preserve the complete anatomy and physiology of the glio-vascular unit. Ideally, these models should integrate fluid mechanics, cell biology, and therapeutic kinetics [35]. The use of velocimetric AI algorithms provides a pathway for non-invasive, high-precision quantification of dynamic CSF parameters in high-dimensional environments, and could contribute to the understanding of clearance mechanisms and tumor progression.

## 7. Conclusions

The intrinsic therapeutic resistance of glioblastoma is a formidable clinical challenge, driven by a combination of fluid-barrier dysfunction and profound immunological disruption. The limited efficacy of chemotherapeutic agents is largely due to the restricted infiltration of drugs across the highly selective BBB, further exacerbated by impairment of the glymphatic system, where tumor mass effect, mis-localization AQP4 in perivascular astrocytic endfeet, and increased intracranial pressure compromise glymphatic function. AQP4 emerges as a central mechanistic axis linking BBB instability, glymphatic impairment, edema, immune evasion, and treatment resistance in GBM. Glymphatic dysfunction manifests as reduced CSF tracer influx and clearance, leading to the accumulation of toxic solutes and pro-inflammatory signaling molecules that promote tumor progression. In addition, reduced CSF outflow through perineural and meningeal lymphatic pathways limits the drainage of tumor-specific antigens to the cervical lymph nodes. This obstruction of antigen drainage results in diminished activation of anti-tumor T cells and a weakened immune response, contributing to the failure of immunotherapy. Therefore, restoring CSF-interstitial communication and lymphatic drainage holds immense therapeutic potential. Modulating glymphatic activity may enhance the delivery of antitumor agents, as perivascular and parenchymal flow can be leveraged to improve antibody transport into the brain. Strategies such as increasing plasma osmolality (e.g., with mannitol or hypertonic saline) and reducing ICP could augment glymphatic influx of intracisternally injected antibodies without BBB disruption. At the same time, restoring immune drainage is crucial; inducing lymphatic remodeling with factors such as VEGF-C has shown to promote antitumor immunosurveillance and enhance the efficacy of immune checkpoint blockade in glioma models.

In the future, the most promising strategy requires an integrated approach. This includes: (1) incorporating advanced imaging, such as DCE-MRI and the DTI-ALPS index, to noninvasively quantify glymphatic dysfunction and provide radiological biomarkers that guide therapeutic planning; (2) using nanomedicine for targeted delivery, such as mesoporous silica nanoparticles (immuno-MSN) loaded with STING agonists, which target perivascular regions rich in antigen-presenting cells to reverse immunosuppression; (3) combining immunotherapy (e.g., ICB or the SMAC mimetic GDC-0152) with fluid-barrier modulation to achieve a synergistic effect in delaying recurrence and improving survival. Certainly, exploiting the glymphatic–immune interface to become a novel and essential translational target for the personalized and effective management of GBM should be an important objective in research and translational efforts.

## Figures and Tables

**Table 1:** Mechanistic Disruptions of the Glymphatic–Immune Axis in Glioblastoma.

Mechanistic Axis	GBM-Induced Alterations	Downstream Pathophysiology	Therapeutic Implications
**Glymphatic flow and CSF-ISF exchange**	Compression of perivascular spaces from tumor mass effect; reduced para-arterial influx and paravenous efflux; decreased bulk flow	Accumulation of toxic solutes, inflammatory cytokines, and metabolic by-products; impaired clearance of tumor-associated molecules	Strategies reducing intracranial pressure, enhancing CSF circulation, and improving solute flux; optimisation of intrathecal delivery exploiting restored flow
**AQP4 localisation and astrocytic endfeet**	AQP4 depolarisation and redistribution across astrocytic membranes; loss of orthogonal arrays of particles; displacement of endfeet by infiltrating tumor cells	Vasogenic edema; destabilised BBB; impaired interstitial transport; reduced glymphatic clearance	AQP4-targeted therapies; osmotherapy to restore fluid gradients; drug strategies leveraging AQP4-dependent CSF-interstitial pathways
**BBB to BTB transition**	Tight junction loss (claudin-5, occludin, ZO proteins); heterogeneous vascular permeability; endothelial activation	Patchy and inconsistent drug delivery; increased extravasation of plasma proteins; permissive microenvironment for invasion	Focused ultrasound mediated BBB opening; intra-arterial mannitol; nanoparticles using receptor mediated transcytosis; improved perfusion-based delivery
**Intracranial pressure and tissue biomechanics**	Increased ICP from mass effect, edema, and vascular leakage; altered interstitial pressure gradients	Reduced intratumoral drug penetration; limited convection-based delivery; impaired CSF flow	ICP modulation, hyperosmolar therapy, and timing of drug administration relative to pressure cycles
**Meningeal lymphatic drainage**	Reduced antigen clearance due to impaired CSF outflow; obstruction by tumor growth; decreased trafficking of APCs to cervical lymph nodes.	Poor T cell priming; weakened systemic antitumor immunity; reduced response to immune checkpoint blockade.	VEGF C-based lymphangiogenesis; photodynamic therapy to enhance lymphatic pumping; strategies improving antigen flow toward lymph nodes
**Pericyte–macrophage–immune crosstalk**	Pericyte hijacking by tumor cells; induction of M2 polarisation via PDGF BB, SOX7, IL pathways; TAM accumulation in hypoxic niches.	Profound immunosuppression; increased IL 10 and TGF beta; impaired cytotoxic infiltration; enhanced tumor progression.	CSF 1R inhibition plus anti fibrotic agents; STING agonists; SMAC mimetics; nanoparticle-based TAM reprogramming.

**Table 2:** Key Imaging and Experimental Tools for Studying Glymphatic and Fluid Dynamics in Glioblastoma.

Tool/Model	Primary Measurement	Strengths	Limitations	Translational Insight
**DCE-MRI**	Vascular permeability, interstitial flow	Widely available; maps BTB leakiness	Indirect for glymphatic flow; contrast required	Identifies invasive margins and predicts response
**DTI-ALPS**	Perivascular diffusivity	Non-invasive; reproducible	Limited assessment of grey matter	Indicates glymphatic dysfunction in GBM
**Intrathecal Contrast MRI**	Direct CSF tracer movement	High anatomical precision	Invasive; long scan times	Visualises lymphatic outflow pathways
**PET CSF Transport**	Solute clearance and efflux	Quantitative kinetics	Limited spatial resolution	Tracks glymphatic transport in vivo
**MRE/Tomoelastography**	Tissue stiffness, viscoelasticity	Reflects biomechanical tumor features	Requires specialised hardware	Links tissue softness with invasion risk
**Orthotopic/GL261 Models**	Tumor–vasculature and immune–fluid interactions	Mechanistic clarity; immune-competent options	Species differences	Dissects GBM effects on glymphatic flow
**Tracer Microscopy**	High-resolution CSF/ISF pathways	Cellular-level detail	Preclinical only	Demonstrates tumor-induced blockage of CSF flow

## Data Availability

No new data were created or analyzed in this study. Data sharing is not applicable to this article.
